# High MUC2 Expression in Ovarian Cancer Is Inversely Associated with the M1/M2 Ratio of Tumor-Associated Macrophages and Patient Survival Time

**DOI:** 10.1371/journal.pone.0079769

**Published:** 2013-12-06

**Authors:** Yi-feng He, Mei-ying Zhang, Xin Wu, Xiang-jun Sun, Ting Xu, Qi-zhi He, Wen Di

**Affiliations:** 1 Shanghai Key Laboratory of Gynecologic Oncology, Department of Obstetrics and Gynecology, Renji Hospital, School of Medicine, Shanghai Jiaotong University, Shanghai, China; 2 Department of Gynecology, Obstetrics and Gynecology Hospital, Fudan University, Shanghai, China; 3 Department of Pathology, Obstetrics and Gynecology Hospital, Fudan University, Shanghai, China; 4 Department of Pathology, First Maternity and Infant Health Hospital, Tongji University, Shanghai, China; Baylor College of Medicine, United States of America

## Abstract

Mucin 2 (MUC2) is a mucin molecule aberrantly expressed by ovarian cancer cells. Previous in vitro studies have indicated that MUC2 promotes cancer growth and metastasis through a tumor-associated macrophage (TAM)-dependent mechanism. However, this mechanism has never been linked to clinical oncology, and its prognostic significance needed to be clarified. Here, we collected 102 consecutive ovarian cancer specimens and used the multiple immuno-histo-chemical/-fluorescent technique to determine the correlations between the MUC2 expression status, the ratio of M1/M2 TAMs and the densities of cyclooxygenase-2 (COX-2)^+^ TAMs and COX-2^+^ cancer cells. The Kaplan-Meier survival analysis and multivariate Cox regression analysis were used to evaluate the prognostic influences of these parameters. As a result, we found that the MUC2 overexpression (immunostaining ++/+++) was significantly correlated with a reduced ratio of M1/M2 TAMs (p<0.001), an increased density of COX-2^+^ TAMs (p<0.001) and an increased density of COX-2^+^ cancer cells (p=0.017). Moreover, most of the M2 TAMs (93%-100%) and COX-2^+^ TAMs (63%-89%) overlapped; and the COX-2^+^ cancer cells were frequently observed near the COX-2^+^ TAMs. In the Cox regression analysis, MUC2 overexpression was found to be an independent prognostic factor for ovarian cancer patients, of which the hazard ratio (HR) was 2.354 (95% confidence interval (CI): 1.031-10.707, p=0.005). Also, the reduced ratio of M1/M2 TAMs and the increased densities of COX-2^+^ TAMs and COX-2^+^ cancer cells were demonstrated to be the predictors of poor prognosis, among which the reduced M1/M2 ratio possessed the highest HR (1.767, 95% CI: 1.061-6.957, p=0.019). All these findings revealed that MUC2 can concurrently exert M2-polarizing and COX-2-inducing effects on TAMs, by which it causes an imbalanced TAM M1-/M2-polarization pattern and induces local PGE_2_ synthesis (in both TAMs and cancer cells). The positive feedback between local PGE_2_ synthesis and TAM M2-polarization accelerates ovarian cancer progression.

## Introduction

Epithelial ovarian cancer threatens the health of adult women and is a leading cause of cancer-related mortality in postmenopausal females [1]. The interactions between ovarian cancer cells and host immune cells have been intensively studied by clinical oncologists to determine how these cancer cells escape or even make use of the host immune system to survive, proliferate and metastasize [2,3]. In previous researches, a series of mucin molecules (MUCs) aberrantly secreted by ovarian cancer cells were identified, including MUC1, MUC2 and MUC16 [4-6]. These mucins comprise a glycoprotein family featuring a serine- and threonine-enriched repetitive polypeptide core and a large number of O-glycans linked to this core [4]. Under physiological circumstances, mucins serve as a protective barrier and lubricant layer that maintains the structure and function of the digestive tract, respiratory tract, reproductive tract and urinary tract, as well as the coeloms, such as the peritoneal cavity, pleural cavity and joint cavities [5]. However, when malignant transformation occurs, the levels of mucin secretion are dramatically enhanced, and the structures of the glycans within these molecules can be altered [7,8]. Once released into the circulation, mucins can serve as cancer biomarkers, such as CA125 (encoded by MUC16) and CA153 (encoded by MUC1) [4-8]. Several preclinical studies have indicated that malignancy-derived mucins can facilitate the progression of cancer through their interactions with immune cells [9-11]. For example, in vitro experiments performed by Inaba et al. showed that MUC2 induced macrophages within cancer tissues to express cyclooxygenase-2 (COX-2) and release prostaglandin E2 (PGE_2_). These authors also suggested that the macrophage-secreted PGE_2_ could in turn promote tumor growth and metastasis [12]. Their findings indicated that MUC2 may be used as an immune suppressor by cancer cells.

The types and numbers of macrophages that infiltrate cancer tissue (i.e., tumor-associated macrophages or TAMs) are closely related to cancer patient prognosis [13,14]. TAMs can be divided into two phenotypes, M1 and M2. M1-polarized TAMs release reactive oxygen and nitrogen intermediates to kill cancer cells or release immunomodulatory factors, such as interleukin-1β (IL-1β) and IL-12, which provoke CD8^+^ T cells to attack cancer cells [13,14]. M2-polarized TAMs have the opposite effects. They release epidermal growth factor (EGF), platelet-derived growth factor (PDGF), tumor transforming growth factor (TGF)-β, vascular endothelial growth factor (VEGF) and other trophic factors that promote cancer cell growth and the cancer vascularization process [13,14]. Moreover, these M2 TAMs can produce a variety of matrix metalloproteinases (MMP2, MMP7, MMP9 and MMP12) and chemokines [C-X-C motif ligand (CXCL) 8, C-C motif ligand (CCL) 13, CCL18 and CCL23] that facilitate cancer micrometastasis [13,14]. Previous studies have shown that a relatively high density of infiltrating M1 TAMs and a high M1/M2 ratio are positively correlated with the five-year survival rate of cancer patients (such as patients with non-small cell lung cancer, prostate cancer and colorectal cancer) [15-17]. In addition, eliminating M2-polarized TAMs using chemotherapeutic agents can inhibit disease progression and improve patient outcome [18,19]. A number of substances released by cancer cells, such as IL4, IL10 and colony-stimulating factor (CSF)-1, have been found to induce monocytes/immature macrophages (M0) to differentiate into M2 TAMs [20,21]. However, the role of mucins secreted by cancer cells in the TAM differentiation process remains unclear.

Because MUC2 has been found to interact with TAMs through their receptors (i.e., the macrophage scavenger receptor) on the cell surface, we were rather interested in whether they can also influence the M1/M2 differentiation of these immune cells. Meanwhile, it is also necessary to interpret the findings of Inaba et al. in a clinically relevant context, which might benefit the clinical treatment and surveillance of ovarian cancer patients. For these purposes, in this study, we quantitatively analyzed the number, density and molecular characteristics of macrophages within ovarian cancer tissues and evaluated the relationships of the obtained TAM parameters with the level of MUC2 expression in cancer tissues. We found that MUC2 played a significant role in the intratumoral TAM differentiation process, favoring the M2 phenotype. Furthermore, based on the findings of Inaba et al.[12], we explored and analyzed the possible COX-2-induction mechanism by which MUC2 mediates the M2 polarization of TAMs and determined the clinical significance of this mechanism for the long-term patient survival.

## Results

### The relationships between the MUC2 expression level and patient demographic, clinical and pathological characteristics

A total of 102 cases were studied (Table 1). Immunohistochemistry demonstrated that the specimens of 47.1% (48 cases) of the enrolled patients exhibited different levels of MUC2 expression (+, ++ or +++). To differentiate the overexpression status of MUC2 from its baseline expression, we performed a parallel immunohistochemical experiment on 33 benign ovarian tumor specimens (methods for collecting these specimens were provided by Materials and Methods S1 in File S1; demographic and pathological characteristics of the 33 patient were provided by Table S1). The results demonstrated “+” MUC2-immunostaining in 36.4% of the benign cases, and no benign tumor cases with higher immunostaining levels were detected (see Figure S1 in File S2). These results suggest that the baseline MUC2 expression in benign ovarian tumors corresponds to MUC2-/+ immunostaining, whereas MUC2++/+++ immunostaining is more specific to malignant tumors and represents a genuine overexpression status [23-25]. We therefore divided the corresponding patients into two groups: a high MUC2 expression group (++ and +++) and a low MUC2 expression group (- and +), with 23 and 79 cases, respectively. Next, we compared the demographic, clinical and pathological characteristics of the patients in the two groups. We found a significant correlation between the MUC2 expression level and the cancer histotype. The rate of MUC2 overexpression was significantly increased in mucinous ovarian cancer (p=0.002, χ^2^ test, Table 1). However, we observed no significant relationships between any of the other parameters, including patient age, body mass index (BMI), gravidity, parity, cancer metastasis status, clinical stage and pathological grade, and the MUC2 expression level (Table 1). 

**Table 1:** Relationships between patient characteristics and MUC2 expression levels *.

Characteristics	Low MUC2 expression group (n=79)	High MUC2 expression group (n=23)	p value**^*†*^**
Demographics:			
Age (years)	59.2±6.1	59.8±6.7	0.680
Gravidity	4.2±1.2	3.6±1.2	0.107
Parity	1.1±0.4	1.2±0.5	0.544
BMI	23.2±2.6	23.0±1.9	0.769
Oral contraceptive use >6 months	7(8.9)	2(8.7)	0.980
Smoking >6 months	12(15.2)	5(21.7)	0.458
Alcohol abuse >6 months	1(1.3)	1(4.3)	0.348
Clinical features:			
Ascites	20(25.3)	7(30.4)	0.624
Peritoneal metastasis (including intestinal, bladder and liver metastasis and peritoneal lavage cytological test +)	62(78.5)	16(69.5)	0.375
Lymph node metastasis	30(38.0)	8(34.8)	0.781
Drug resistance during the primary chemotherapeutic course	2(2.5)	1(4.3)	0.650
Pathology:			
Stage ^**^			0.383
II	23(29.1)	4(17.4)	
III	53(67.1)	17(73.9)	
IV	3(3.8)	2(8.7)	
Histotype			0.002**^*§*^**
Serous	57(72.2)	10(43.5)	
Mucinous	7(8.9)	10(43.5)	
Endometrioid	8(10.1)	1(4.3)	
Clear cell	5(6.3)	2(8.7)	
Undifferentiated	2(2.5)	0(0)	
Grade ^**^			0.182
G1	40(50.6)	8(34.8)	
G2	19(24.1)	10(43.5)	
G3	20(25.3)	5(21.7)	

* Data are presented as the mean values±standard deviation or as the number (percentage)

^**^ See reference 22

^†^ A two-tailed Student’s t-test or χ^2^ test was used, as appropriate.

^§^ Statistical significance.

### Correlation between the ovarian cancer tissue MUC2 expression status and the TAM M1/M2 ratio

**Figure 1:**
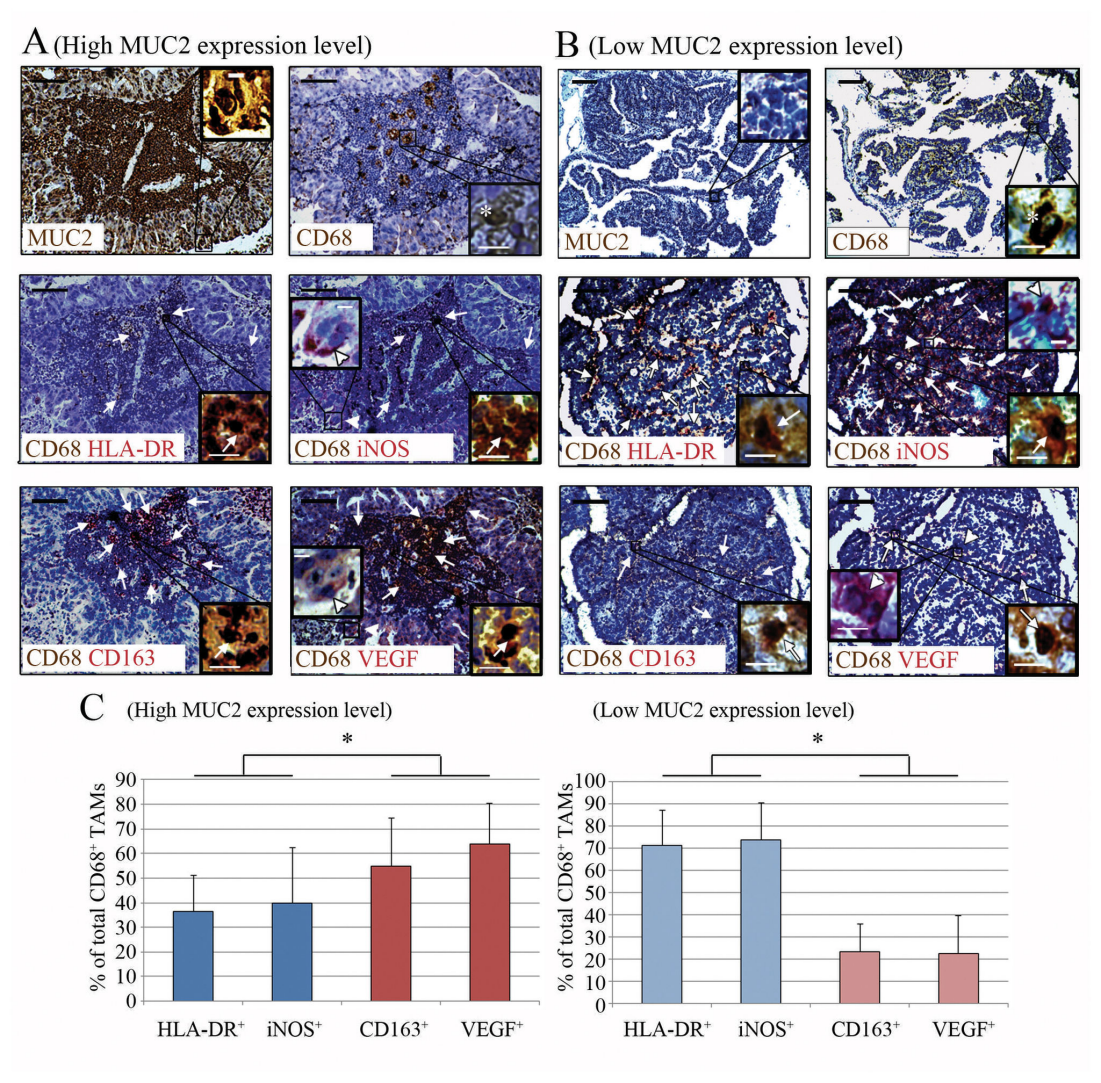
Total CD68^+^ TAMs and their M1 and M2 subsets in the ovarian cancer tissue. (**A**) Representative MUC2 and CD68 single-immunostained and CD68/HLA-DR, CD68/iNOS, CD68/CD163, CD68/VEGF double-immunostained sections from the high MUC2 expression group. (**B**) Representative MUC2 and CD68 single-immunostained and CD68/HLA-DR, CD68/iNOS, CD68/CD163, CD68/VEGF double-immunostained sections from the low MUC2 expression group. For VEGF- and iNOS-immunostaining, cancer cells (in red) that independently expressed these two proteins can be found in the cancer tissue sections from both groups (arrowhead). Asterisk, TAMs (in brown) that expressed the CD68 protein. Arrow, CD68^+^ TAMs (in purple) that co-expressed the indicated proteins (HLA-DR, iNOS, CD163 or VEGF). Scale bar (black), 100 μm. Inset scale bar (white), 10 μm. (**C**) Comparison of the percentages of different M1 and M2 cell subsets (i.e., M1/M2 distribution patterns) among all the TAMs. It could be noted that the percentages of M1 macrophages were significantly higher than those of M2 macrophages in the high MUC2 expression group; but on the contrary, the percentages of M1 macrophages were significantly lower than those of M2 macrophages in the low MUC2 expression group. *, p<0.05, ANOVA.

The TAMs in the ovarian cancer specimens were quantitatively analyzed based on CD68 single immunostaining [17,26]. The densities of CD68^+^ cells in the cancer tissues from the high MUC2 expression group and the low MUC2 expression group were similar (27.3±13.6 mm^-2^ vs. 26.0±11.9 mm^-2^); and no statistical significance was established (p=0.654, Student’s t test). Next, the cancer specimens were double immunostained to explore the intratumoral differentiation status of the TAMs. The anti-CD68 + anti-human leukocyte antigen (HLA)-DR and anti-CD68 + anti-inducible nitric oxide synthase (iNOS) antibody panels were used to identify M1-polarized TAMs, and the anti-CD68 + anti-CD163 and anti-CD68 + anti-VEGF antibody panels were used to identify M2-polarized TAMs (Figure 1) [17,26]. Our preliminary test confirmed that HLA-DR^+^CD163^+^, HLA-DR^+^VEGF^+^, iNOS^+^CD163^+^ and iNOS^+^VEGF^+^ TAMs accounted for less than 3% (1.2%, 1.7%, 2.1% and 2.6%, respectively) of the total TAMs, indicating that the TAMs are scarcely double labeled with both M1 and M2 signatures. Upon examining the cancer tissue sections, we observed that the intra-islet density of M1-polarized TAMs (CD68^+^ HLA-DR^+^ or CD68^+^ iNOS^+^) was significantly lower in the high MUC2 expression group than in the low MUC2 expression group (p<0.01, Student’s t test, Table 2). In contrast, the intra-islet density of M2-polarized TAMs (CD68^+^ CD163^+^ or CD68^+^ VEGF^+^) in the high MUC2 expression group was significantly higher than that of the low MUC2 expression group (p<0.001, Student’s t test, Table 2). Furthermore, we analyzed the M1/M2 ratios based on TAMs staining with CD68^+^ HLA-DR^+^ or CD68^+^ CD163^+^ in two consecutive tumor sections from each patient. The resulting mean M1/M2 ratio was significantly lower in the high MUC2 expression group than in the low MUC2 expression group (p<0.001, Student’s t test, Table 2), which was consistent with the M1/M2 distribution patterns of the TAMs of these two groups (Figure 1). 

**Table 2:** Density and M1/M2 ratio of TAMs within the islet region of the ovarian cancer tissue.

Groups	Total CD68^+^ TAM density^*^	M1 density^*^ (HLA-DR)	M1 density^*^ (iNOS)	M2 density^*^ (CD163)	M2 density^*^ (VEGF)	M1/M2 ratio (calculated)^†^	M1/M2 ratio (actual)^†^
High MUC2 expression group	17.4±8.3	6.7±5.8	6.6±4.7	9.9±7.2	11.5±7.9	0.69±0.28	0.58±0.12
Low MUC2 expression group	16.1±8.2	11.0±7.2	12.0±7.3	4.6±3.9	4.5±3.8	2.75±2.42	2.83±2.16
p value^**^	0.523	0.008^§^	0.001^§^	<0.001^§^	<0.001^§^	<0.001^§^	<0.001^§^

* Data are presented as the mean values±SD per mm^2^. The total TAM density was determined by CD68 single immunostaining, and the M1 and M2 TAM densities were determined using CD68-based double immunostaining. The signatures used to identify the M1 and M2 phenotypes of TAMs are indicated in parentheses.

** Two-tailed Student's t test.

^†^ The M1/M2 ratio was calculated by dividing the M1 TAM density (CD68 and HLA-DR-positive) by the M2 TAM density (CD68 and CD163-positive) in two representative sections^.17,23^ The actual M1/M2 ratio is defined as the number of CD68^+^ HLA-DR^+^ cells divided by the number of CD68^+^ CD163^+^ cells in the same fields of two consecutive sections.^17,23^

^§^ Statistical significance.

### Correlation between the ovarian cancer tissue MUC2 expression status and TAM COX-2 expression

**Figure 2:**
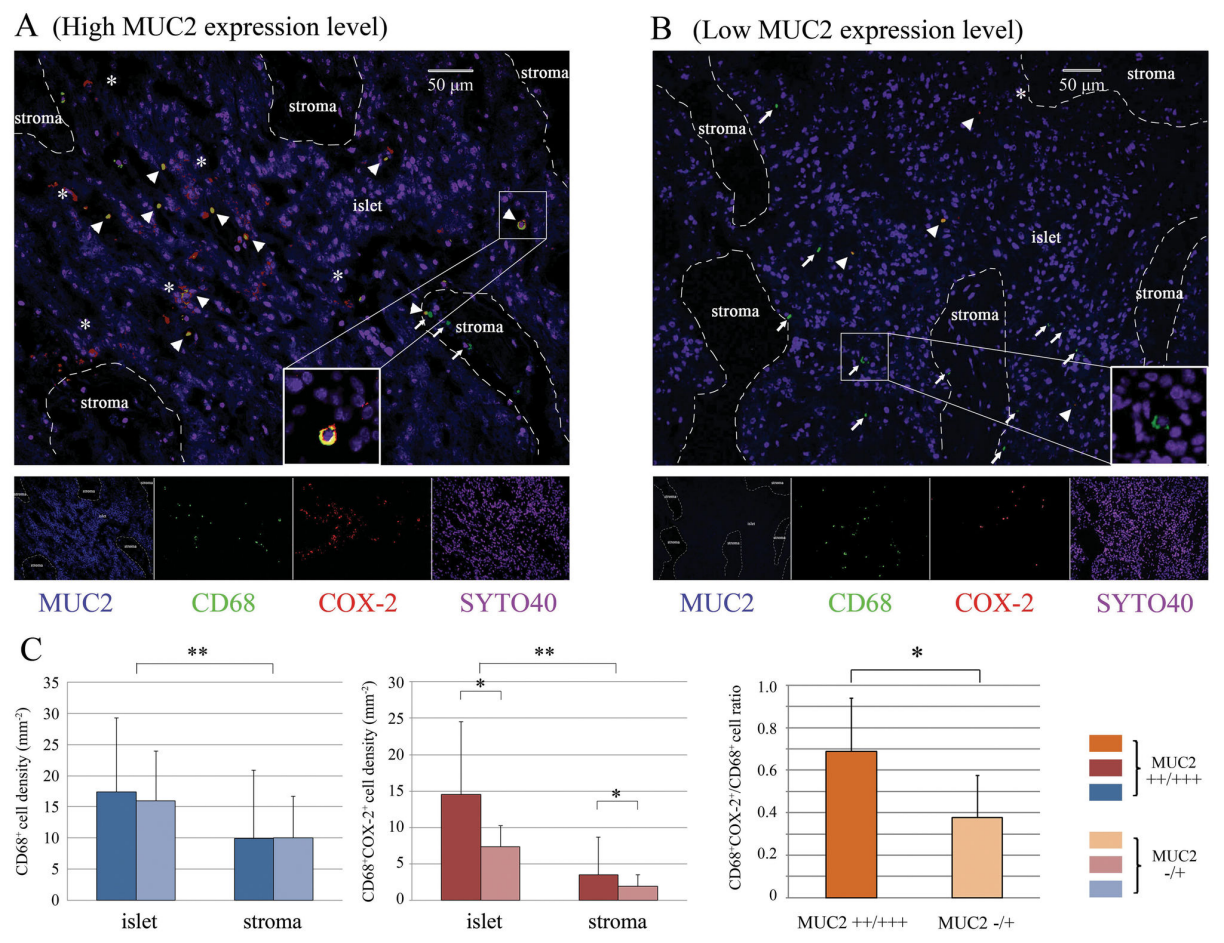
Distribution characteristics of CD68^+^COX-2^+^ TAMs in the ovarian cancer tissue. (**A**) CD68^+^COX-2^+^ TAMs in a representative cancer tissue section from the high MUC2 expression group were shown. (**B**) CD68^+^COX-2^+^ TAMs in a representative cancer tissue section from the low MUC2 expression group were shown. Nuclei were stained with SYTO 40, shown in purple. Arrowhead, CD68^+^COX-2^+^ TAMs. Arrow, CD68^+^COX-2^-^ TAMs. Asterisk, COX-2^+^ cancer cells (CD68^-^) surrounding the CD68^+^COX-2^+^ TAMs. (**C**) The intratumoral densities of CD68^+^ TAMs and CD68^+^COX-2^+^ TAMs and the CD68^+^COX-2^+^/CD68^+^ TAM ratios of the high and low MUC2 expression groups were compared, as were the corresponding values for the cancer islet and stromal regions. It could be found that the ratio of CD68^+^COX-2^+^/CD68^+^ TAMs was significantly higher in the high MUC2 expression group than in the low MUC2 expression group *, p<0.05, Student’s t-test. **, p<0.05, ANOVA.

To establish the relationship between the expression status of MUC2 in cancer tissue and COX-2 expression level in TAMs we compared the densities of COX-2^+^ TAMs in cancer specimens between the high and low MUC2 expression groups. As shown in Figure 2, we found that, regardless of the overall MUC2 expression level, the density of CD68^+^COX-2^+^ TAMs was significantly higher in the cancer islets, in which MUC2 molecules were vigorously secreted, than in the stromal regions, in which MUC2 molecules were scarcely secreted (p<0.001, ANOVA, Figure 2). Moreover, either in the cancer islets or in the stromal regions, the density of CD68^+^COX-2^+^ TAMs was significantly higher in the high MUC2 expression group than in the low MUC2 expression group (p<0.001, ANOVA, Figure 2). Additionally, in the whole section (cancer islet+stromal region), the mean ratio of CD68^+^COX-2^+^ TAMs/total CD68^+^ TAMs was significantly higher in the high MUC2 expression group than in the low MUC2 expression group (p<0.001, Student’s t test, Figure 2). A number of CD68^-^COX-2^+^ cancer cells were observed surrounding the CD68^-^COX-2^+^ TAMs, particularly in the specimens from the high MUC2 expression group (Figure 2). 

### M1 and M2 differentiation patterns and their relationship with TAM COX-2 expression

**Figure 3:**
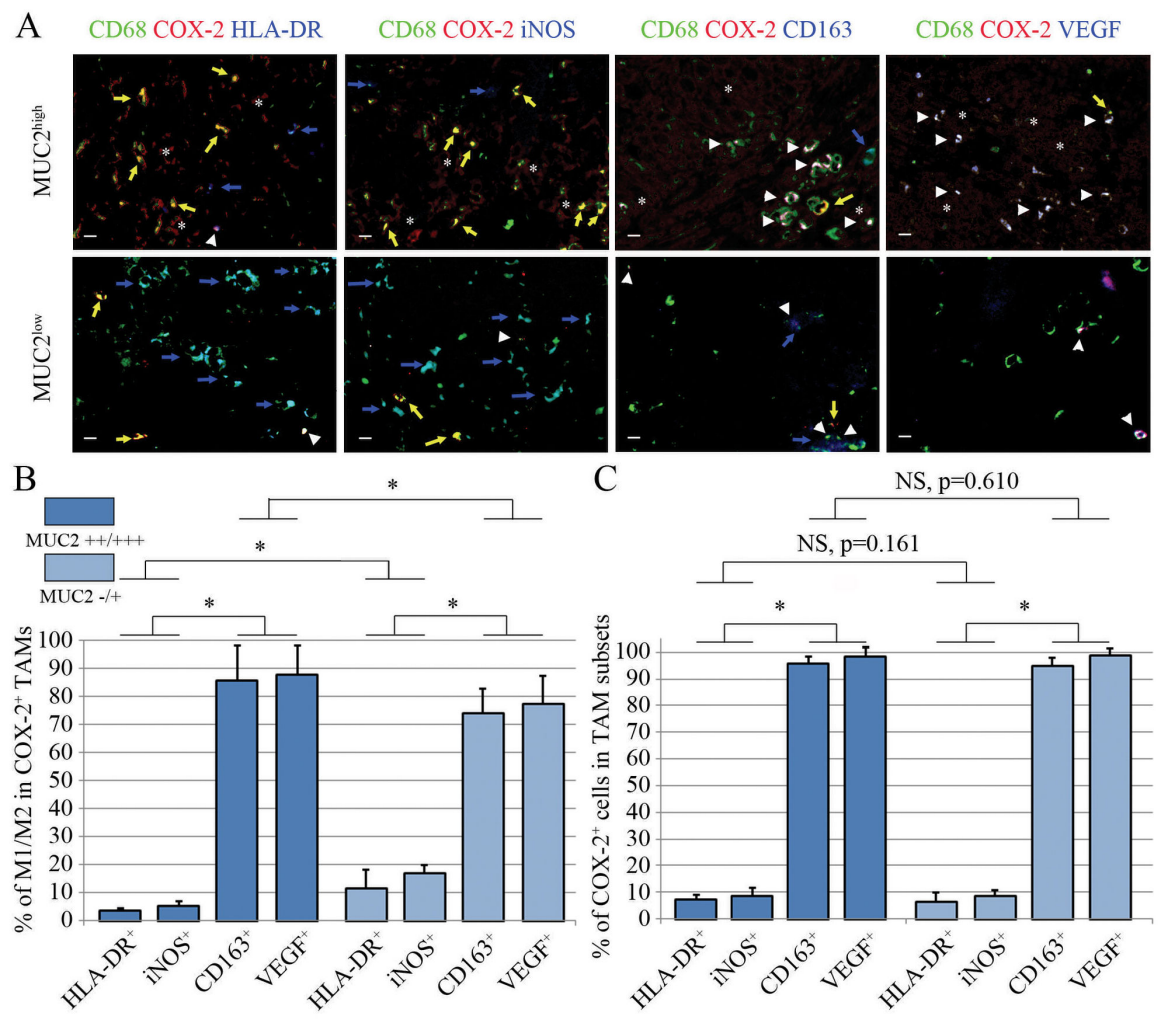
M1 and M2 distribution patterns of the COX-2^+^ TAMs in the high and low MUC2 expression groups. (**A**) Representative CD68, COX-2 and M1/M2 signature triple-immunostained sections are shown. Arrowhead, COX-2^+^ TAMs that expressed the M1 or M2 signatures. Yellow arrow, COX-2^+^ TAMs that did not express the M1 or M2 signature indices. Blue arrow, COX-2^-^ M1 or M2 TAMs. Asterisk, COX-2^+^ cancer cells. Scale bar, 10 μm. (**B**) The percentages of M1- and M2-polarized TAMs in the CD68^+^COX-2^+^ TAM populations that infiltrated the cancer tissue in the two MUC2 expression-level groups were compared. (**C**) The percentages of COX-2^+^ cells in the M1 and M2 populations of the intratumoral TAMs in the two MUC2 expression-level groups were compared. It could be noted that most of the COX-2^+^ TAMs in both the high and low MUC2 expression groups exhibited M2 signatures (i.e., CD163^+^ and VEGF^+^). *, p<0.05, ANOVA. NS, no significance, ANOVA.

To establish the relationship between the COX-2 expression level and the differentiation status of the TAMs, we used a triple-immunostaining technique to determine the M1- and M2-differentiation patterns of the CD68^+^COX-2^+^ TAMs that had infiltrated the ovarian cancer tissues. Generally, in all specimens, 3%-20% of the CD68^+^COX-2^+^ TAMs were identified as M1 polarized (HLA-DR^+^ or iNOS^+^), and 63%-89% were identified as M2 polarized (CD163^+^ or VEGF^+^), regardless of the MUC2 expression status. However, the mean percentage of M1-polarized COX-2^+^ TAMs was significantly lower in the high MUC2 expression group than in the low MUC2 expression group (p<0.001, ANOVA); and the mean percentage of M2-polarized COX-2^+^ TAMs was significantly greater in the high MUC2 expression group (p<0.001, ANOVA, Figure 3). In addition, the COX-2^+^ cells in the cancer tissue sections accounted for 3%-13% of all the observed M1 TAMs (i.e., CD68^+^ HLA-DR^+^ or CD68^+^ iNOS^+^ cells), and accounted for 93%-100% of the M2 TAMs observed (CD68^+^ CD163^+^ or CD68^+^ VEGF^+^). The ratios of COX-2^+^ cells in the M1 and M2 TAM populations of the two MUC2 expression groups were not significantly different (for M1 subsets, p=0.161 and for M2 subsets, p=0.610, ANOVA, Figure 3). 

### Correlation between MUC2 expression status and COX-2 expression status in ovarian cancer tissue

**Figure 4:**
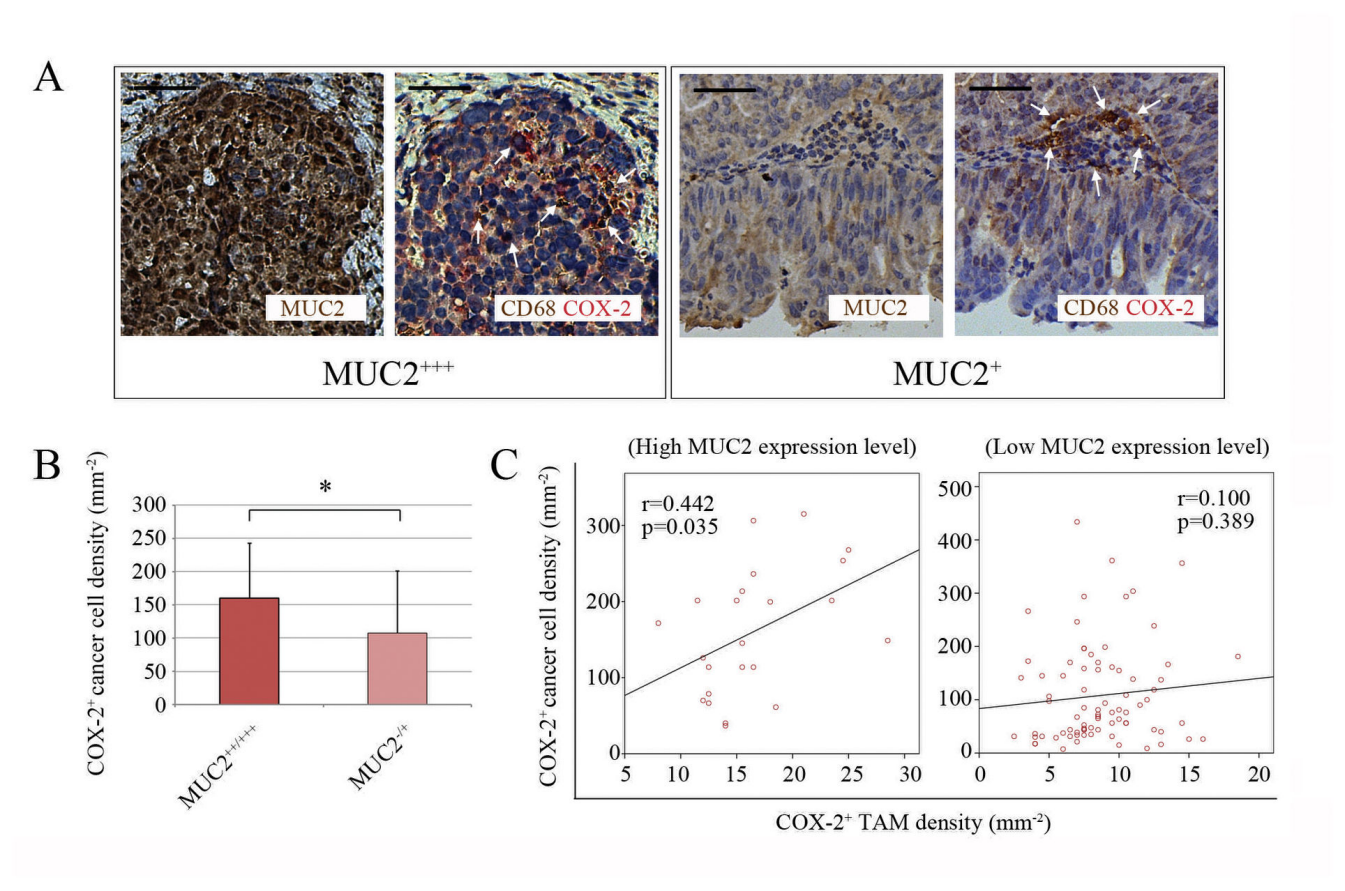
Correlation of the COX-2 expression statuses of the cancer cells and TAMs. (**A**) Representative microscopic fields in cancer tissue sections from the high and low MUC2 expression groups. In the cancer tissue sections from the high MUC2 expression group, COX-2^+^ cancer cells and COX-2^+^ TAMs (with cytoplasm in red) were frequently distributed throughout the entire islet region, whereas in the cancer tissue from the low MUC2 expression group, COX-2^+^ cancer cells and COX-2^+^ TAMs were both observed less frequently. Arrow, CD68^+^ TAMs (left, CD68^+^COX-2^+^ TAMs in purple; right, CD68^+^COX-2^-^ TAMs in brown). Scale bar, 50 μm. (**B**) Comparison of the COX-2^+^ cancer cell densities between the high and low MUC2 expression groups. *, p<0.05, Student’s t-test. (**C**) Scatter plots of the COX-2^+^ cancer cell density versus the COX-2^+^ TAM density in specimens from the high (23 cases) and low MUC2 expression (79 cases) groups. r, Pearson’s product-moment correlation coefficient.

It was previously reported that PGE_2_, the catalytic product of COX-2 released by COX-2^+^ TAMs can upregulate the intracellular expression of COX-2 in the cultured cancer cells [27]. Accordingly, we investigated the relationship between the COX-2 expression statuses of ovarian cancer cells and the infiltrating TAMs in the enrolled patient population. The obtained data showed that the density of COX-2^+^ cancer cells was significantly higher in the high MUC2 expression group (p=0.017, Student’s t test, Figure 4), with a significantly increased local concentration of PGE_2_ detected using the enzyme-linked immunosorbent assay (ELISA) (methods for performing ELISA were provided by Materials and Methods S2 in File S1, the results of the PGE_2_ ELISA experiment were provided by Figure S2 in File S2) compared to the low MUC2 expression group. Moreover, a statistically significant correlation was found between the density of COX-2^+^ cancer cells and that of COX-2^+^ TAMs in the specimens from the high MUC2 expression group, and most of the COX-2^+^ cancer cells were detected in the regions near the COX-2^+^ TAMs (p=0.035, Pearson’s correlation analysis, Figure 4). However, no such correlation was found in the low MUC2 expression group, suggesting that the majority of the COX-2^+^ cancer cells observed in the ovarian cancer specimens of this group most likely resulted from spontaneous gene expression, but not the expression induced by a few neighboring COX2^+^ TAMs (p=0.389, Pearson’s correlation analysis, Figure 4).

### Kaplan-Meier survival analysis and multivariate Cox regression analysis of patient outcomes

**Figure 5:**
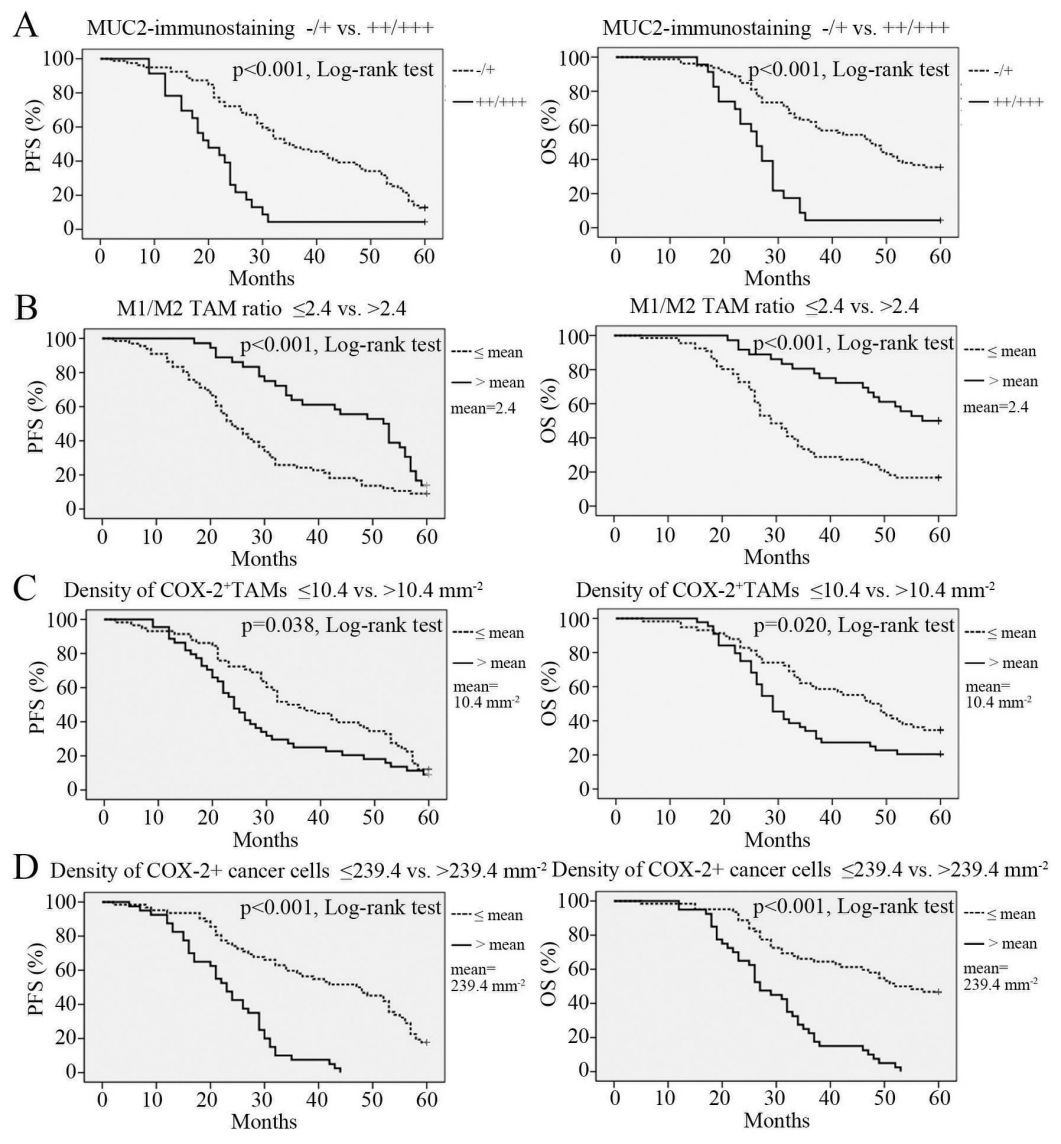
Kaplan–Meier 5-year PFS and OS curves for ovarian cancer patients stratified by four pathological parameters of interest. (**A**) MUC2 expression level, low (immunostaining -/+) vs. high (immunostaining ++/+++). (**B**) M1/M2 ratio, lower vs. higher than the mean value. (**C**) COX-2^+^ TAM density, lower vs. higher than the mean value. (**D**) COX-2^+^ cancer cell density, lower vs. higher than the mean value. PFS, progression-free survival. OS, overall survival.

Since we observed that the MUC2 expression level affected the M1/M2 ratio of the TAMs in the evaluated cancer tissues, we further explored whether this local immunological change could exert a substantial impact on the 5-year survival of the enrolled cancer patients. We performed a Kaplan-Meier survival analysis and a multivariate Cox regression analysis to examine the relationship between the MUC2 expression level and patient survival. The reference parameters used in the two analytical methods included patient age, BMI, ascites status and metastasis, as well as cancer stage, histotype and grade, the size of residual site, the M1/M2 ratio of the TAMs and the COX-2 expression levels of the TAMs and cancer cells. The Kaplan-Meier survival analysis showed that the 5-year progression-free survival (PFS) rate and the overall survival (OS) rate were both significantly lower in the high MUC2 expression group than in the low MUC2 expression group (p<0.001 for PFS and p<0.001 for OS, log-rank test, Figure 5). In addition, a reduced M1/M2 ratio and increased densities of COX-2^+^ TAMs and COX-2^+^ cancer cells were both the predictors of poor prognosis (Figure 5, for prognostic significance of other parameters, see Figure S3 in File S2). The multivariate Cox regression analysis showed that the level of MUC2 expression in the cancer cells, peritoneal metastasis, cancer stage, histotype and grade, the size of residual site, the M1/M2 ratio of the TAMs and the densities of COX-2^+^ TAMs and COX-2^+^ cancer cells were independent prognostic factors for ovarian cancer patient outcomes (Table 3). 

**Table 3:** Multivariate Cox regression analysis of potential prognostic factors for ovarian cancer.

Parameter*	Hazard ratio^†^	95% confidence interval	p value
Age > 59.1 years	0.991	0.939-1.045	0.731
BMI > 23.2	0.987	0.880-1.108	0.830
Existence of ascites	0.991	0.472-1.819	0.824
Existence of peritoneal metastasis	2.248	1.126-5.629	0.002^§^
Existence of lymphatic metastasis	1.226	0.607-2.477	0.570
Size of residual site >2 cm	3.073	1.240-7.223	<0.001^§^
Stage			0.004^§^
III	2.556	1.128-9.036	
IV	5.936	1.057-16.078	
Histotype			0.016^§^
Mucinous	1.073	1.075-3.969	
Endometrioid	0.750	1.571-5.394	
Clear cell	1.055	1.334-7.881	
Undifferentiated	2.345	1.450-12.216	
Grade			0.015^§^
G2	1.901	1.445-7.827	
G3	2.381	1.785-9.788	
Density of CD68^+^COX-2^+^ TAMs > 10.4 mm^-2^	1.010	1.006-1.035	0.042^§^
Density of CD68^-^COX-2^+^ cancer cells > 239.4 mm^-2^	1.006	1.002-1.009	0.002^§^
TAM M1/M2 ratio ≤ 2.4	1.767	1.061-6.957	0.019^§^
MUC2-immunostaining ++/+++	2.354	1.031-10.707	0.005^§^

* To be concise, the reference groups (hazard ratio=1) for each parameter were omitted. These groups were “Age ≤ 59.1 years”, “BMI ≤ 23.2”, “No ascites”, “No peritoneal metastasis”, “No lymphatic metastasis”, “Size of residual site ≤ 2 cm”, “Stage II’, “Serous”, “G1”, “Density of CD68^+^COX-2^+^ TAMs ≤ 10.4 mm^-2^”, “Density of CD68^-^COX-2^+^ cancer cells ≤ 239.4 mm^-2^”, “TAM M1/M2 ratio > 2.4” and “MUC2-immunostaining -/+”, respectively. All the patients accepted a standard taxol + platinum therapy, therefore, the chemotherapy scenario was not included as a parameter for the multivariate analysis.

^†^ The hazard ratio was defined as the rate of patient death in the target group divided by the rate of patient death in the reference group during the 5-year follow-up.

^§^ Statistical significance.

## Discussion

To our knowledge, this study is the first to investigate the actual immunomodulatory effect of the MUC2 molecules secreted by ovarian cancer cells based on a molecular pathology approach, which provided new insight into the relationship between cancer cells and TAMs. MUC2 overexpression has repeatedly been demonstrated as a poor prognostic factor for non-digestive system cancers, such as breast cancer, bladder cancer and ovarian cancer, in previous studies [28-30]. However, the detailed mechanism by which it promotes cancer progression has not been adequately investigated. In this population-based analysis, we discovered a series of cancer progression-promoting changes (i.e., elevated levels of CD163, VEGF and COX-2 expression) in the TAMs that contacted the MUC2^++/+++^ ovarian cancer tissue and a number of complex and distinctive interactions between the cancer cells and the defending macrophages were identified. 

 As shown in Figure 1 and Table 2, only the M1/M2 ratio of the TAMs differed between the high and low MUC2 expression groups, whereas the TAM densities in these two groups were similar. This phenomenon suggests that MUC2 is only involved in the process of TAM differentiation, not in the process of TAM recruitment. This immunomodulatory effect is different from that of many known cytokines that possess both TAM differentiation-inducing and chemotactic effects, such as VEGF, CSF-1, CCL2/3/5 and glypican-3 (GPC3) [31-34], and may arise because the MUC2 receptor (i.e., macrophage scavenger receptor) is not coupled to the cellular adhesion and motility units in the monocytes/macrophages [35]. The current opinions regarding the prognostic impact of the altered M1/M2 ratio induced by MUC2 expression are somewhat contradictory. Ohri et al. indicated that a higher M1/M2 ratio led to an increased 5-year survival rate in non-small cell lung cancer patients, but they also found that the altered M1/M2 ratio were resulted from the significantly increased infiltration of M1 TAMs (with less increased infiltration of M2 TAMs) and was therefore not the result of an imbalanced TAM polarization process [15]. Later, Edin et al. indicated that it was the number of infiltrating M1 TAMs, rather than the change in M1/M2 ratio, that substantially influenced the prognosis of colorectal cancer patients [16]. However, a study conducted by Zhang et al. found the opposite. The authors observed that an increased M1/M2 TAM ratio alone was sufficient to predict a better prognosis for lung cancer patients [36]. Soon thereafter, Algars et al. corroborated the finding that it was the M1/M2 ratio itself, rather than altered densities of the infiltrating M1 or M2 TAMs, that predicted a reduced long-term incidence of cancer relapse or hepatic metastasis in patients with colorectal cancer [37]. Therefore, our results validated the findings of Zhang et al. and Algars et al. We demonstrated that an altered M1/M2 ratio alone is an independent prognostic indicator for ovarian cancer patients (Table 3, Figure 5), which also implies that the chemotactic effect on monocytes/macrophages is not necessary for some immunosuppressive factors, such as MUC2, to influence patient prognosis. Moreover, considering that the reduced M1/M2 ratio has the highest HR (Table 3), this pathological factor might have played a major role in the adverse outcome of MUC2^++/+++^ ovarian cancer cases. 

 It was previously reported that cancer-derived MUC2 could initiate intracellular signaling by binding to the macrophage scavenger receptor (MSR1) on the surface of infiltrating monocytes/macrophages, promoting the upregulation of intracellular COX-2 gene expression in these cells [12]. In this study, we re-examined this hypothesis in 102 cancer specimens. We found that most of the TAMs, which were closely surrounded by MUC2^+^ cancer cells, exhibited upregulated COX-2 expression (i.e., an increased ratio of CD68^+^COX-2^+^ cells/total CD68^+^ cells in the cancer islets, see Figure 2). Moreover, we noted that this phenomenon was particularly significant in the high MUC2 expression group (Figure 2). These findings indicate that the local MUC2 expression level can help to determine the intratumoral density of COX-2^+^ TAMs, which implies its clinical significance. It has been known that COX-2 overexpression is associated with the increased synthesis of PGE_2_ in macrophages [38]. The release of PGE_2_, which is a proinflammatory factor, can induce increases in the expression of VEGF, MMP, multi-drug resistance 1 (MDR1) and B-cell lymphoma 2 (Bcl-2) in the surrounding cancer cells, resulting in an improved vascularization of the cancer tissue and a reduced rate of apoptosis as well as an enhanced rate of metastasis and drug resistance in the cancer cell population [39]. Hence, the existence of PGE_2_-releasing TAMs is generally an unfavorable prognostic factor [40]. Because PGE_2_ is easily degraded in the immunohistochemical labeling process [41], we had to immunohistochemically examined the COX-2 expression status in TAMs instead and used the obtained data for completing a Kaplan-Meier analysis. The result indicated that the COX-2 overexpression of TAMs was indeed associated with poor prognosis in the enrolled patients (Figure 5), suggesting that MUC2 also impaired patient survival via altering the local density of COX-2^+^ TAMs. 

Interestingly, in our study, TAMs with high COX-2 expression were predominantly M2 polarized, and vice versa; the majority of M2 TAMs in the MUC2^++/+++^ cancer tissues were COX-2^+^ (Figure 3). Moreover, we observed that an increased density of COX-2^+^ TAMs was associated with a poor patient prognosis, consistent with the prognostic value of the increased proportion of M2 TAMs (Figure 5, see Kaplan-Meier curves for COX-2^+^ TAM density and M1/M2 TAM ratio, respectively). Based on these two findings, it is reasonable to conclude that the overexpression of COX-2 is an alternatively activated characteristic of M2 TAMs. However, these data raised a critical question: what is the mechanism through which COX-2^+^ TAMs are directed toward an M2 phenotype? A literature search revealed that PGE_2_, which is the catalytic product of COX-2, might participate in the differentiation of intratumoral monocytes/macrophages. In a seminal study, Torroella-Kouri et al. demonstrated that PGE_2_ could downregulate the transcriptional activity of NF-κB in immature monocytes and macrophages, which in turn reduces the expression levels of key M1-phenotype genes, such as iNOS and IL12 [42]. In a subsquent study, Heusinkveld et al. found that an elevated level of PGE_2_ in the local microenvironment inhibited the differentiation of TAMs into the M1 phenotype and promoted their polarization into the M2 phenotype [43]. Recently, Nakanishi et al. confirmed that the administration of celecoxib, a selective COX-2 inhibitor, induced M2-polarized TAMs to become M1-polarized TAMs [44]. Taken together, these data indicate that PGE_2_ expression in the tumor microenvironment can guide undifferentiated monocytes and TAMs to enter the M2-polarization pathway. In our study, a high level of MUC2 expression in cancer cells was found to be correlated with a significantly increased level of COX-2 expression in TAMs and accompanied by a greatly elevated level of PGE_2_ in the local tissue (see Figure S2 in File S2). While residing in this PGE_2_-enriched microenvironment, immature monocytes/macrophages could be inevitably polarized to the M2 phenotype (of course, we did not exclude the activities of other pathways that might mediate the M2 polarization of COX-2^+^ TAMs; further investigation is needed); besides, the short-range dispersion of PGE_2_ could also induce M2 differentiation in nearby monocytes/macrophages that had no direct contact with MUC2^+^ cancer cells (Figure 2, see the COX-2^+^ TAMs grouped in the stromal region where MUC2 is weakly expressed). This paracrine-style macrophage-differentiation induction mechanism could be an important pathway by which MUC2 alters the M1/M2 ratio of TAMs. Together with the additionally released M2-type cytokines (e.g., VEGF, PDGF and EGF) [13,14], this mechanism can augment the original cancer-promoting effect of the COX-2^+^ TAMs (Table 3). 

 During this study, we have also attempted to correlate the MUC2 expression level with the intracellular expression of COX-2 in MUC2^+^ ovarian cancer cells, as indicated in Figure 4. We observed that an elevated MUC2 level in ovarian cancer tissues was often accompanied by a significant upregulation of COX-2 expression in ovarian cancer cells (accompanied by an upregulation of the PGE_2_ concentration, see Figures 2 and 4 and Figure S2 in File S2). We further demonstrated that the induction of COX-2 overexpression in cancer cells is an independent predictor of poor patient outcome (Table 3). Considering that the upregulation of COX-2 expression in the TAMs and MUC2^+^ cancer cells was largely concomitant and consistent (Figure 4), we speculated that the regulation of COX-2 expression in these two cell types could be inherently linked. We noted that the findings of Sonoshita et al. could help to explain the mechanism underlying this phenomenon. These authors reported that PGE_2_ binds to the prostaglandin E2 (EP2) receptors of cancer cells, which upregulates the intracellular transcription of COX-2 after EP2 activation, thereby creating a positive feedback loop leading to the dramatically increased expression of COX-2 [27]. Moreover, exogenous PGE_2_ molecules can also initiate this positive feedback loop. Therefore, a small amount of exogenous PGE_2_, such as the PGE_2_ released by a few COX-2^+^ TAMs, can trigger a strong response in surrounding cancer cells, causing them to express COX-2. Given this mechanism, we suggested that the following molecular functional chain could exist in ovarian cancer tissue: MUC2 (ovarian cancer cells) → MSR1 (TAMs) → COX-2 (TAMs) → PGE_2_ (TAMs) → EP2 (ovarian cancer cells) → COX-2 (ovarian cancer cells) → PGE_2_ (ovarian cancer cells). This pathway allows PGE_2_, a recognized cancer progression-accelerating factor, to rapidly accumulate in the local tissue microenvironment (see Figure S2 in File S2), eventually leading to an adverse outcome. In the context of this condition, we concluded that COX-2^+^ cancer cells could be a critical co-factor in the poor prognosis caused by MUC2 overexpression in addition to MUC2-induced COX-2^+^ and M2 TAMs. 

Regarding the cancer histotype, we observed that mucinous ovarian cancer most frequently expressed MUC2 molecules. The rate of MUC2 overexpression was 58.8% (10/17, see Table 1) in the mucinous ovarian cancer cases, which was significantly higher than the rates of 14.9% (10/67), 12.5% (1/8), 40% (2/5) and 0% (0/2) in the serous, endometrioid, clear cell and undifferentiated cancer cases, respectively (Table 1). This expression pattern is consistent with that reported by Feng et al [45]. The authors found that MUC2 was expressed almost exclusively in mucinous ovarian cancers in the specimens they examined. Considering that MUC2 molecules are always expressed by the glandular cells of the digestive tract under normal physiological conditions, we postulated that the overexpression of MUC2 in mucinous ovarian cancer cells might reflect a histological origin similar to that of gastrointestinal epithelial cells. In addition, we did not find any correlation between the level of MUC2 expression and the clinical stage/pathological grade of the cancers, which is also consistent with the findings of Feng et al [45]. Although Dong et al. stated that MUC2 molecules were more frequently expressed in low-grade mucinous ovarian cancer samples [46], our study showed only a trend that MUC2 was overexpressed in G2 cases (16% for G1, 34% for G2, and 20% for G3, p=0.182, χ^2^ test, Table 1). Future studies that involve larger populations and/or compare more strains of different anti-MUC2 monoclonal antibodies may be helpful to address this problem. Serous and mucinous ovarian cancers are the two most common histotypes of ovarian cancer encountered in the clinic [1]. Previous studies have indicated that among patients with ovarian cancer of advanced stages with similar clinical stages and pathological grades, those with a serous histotype have a longer survival time than those with a mucinous histotype [47,48]. The patients enrolled in our study had ovarian cancers that were mostly at stages IIb-IIIc (95%, see Table 1), and the Kaplan-Meier analysis indicated that the 5-year survival rates (both PFS and OS) for patients with mucinous cancer were significantly lower than those for patients with serous/endometrioid cancer (see Figure S3 in File S2). Therefore, our results support the previous clinical findings. Because MUC2 molecules are frequently expressed in mucinous cancers, we hypothesized that the secreted MUC2 molecules allow cancer cells (with the elevated level of PGE_2_) to more easily survive chemotherapy. This may explain our observation that the patients with mucinous ovarian cancer experienced a higher risk of cancer relapse than did those with serous cancer (see Figure S3 in File S2). 

One limitation of this study is that, due to its observational nature, we cannot determine whether PGE_2_ induction by MUC2 overexpression is the only pathway through which MUC2 disrupts the M1/M2 polarization balance of TAMs. Given that MSR1, the MUC2 receptor, can also mediate many other bioactivities of monocytes/macrophages [49-51], the direct M2-polarizing effect of MSR1 should not be neglected. We are currently using an in vitro MUC2-based M2-polarization experiment in which COX-2 expression has been knocked down (using small interfering RNAs) in TAMs to evaluate the independent effect of MSR1. Our preliminary results indicate that M2 polarization is significantly decreased, but not abolished, in TAMs treated with MUC2 molecules (data not shown), suggesting that other, less influential, pathways allow activated MSR1 to mediate the M2-polarizing effect. Nevertheless, the results of this experiment did not conflict with our current conclusions regarding the roles of MUC2 and PGE_2_ because COX-2 was required for the full M2-polarizing effect of MUC2. Further experiments are underway to investigate the intracellular pathways that mediate the M2-polarizing effect of MSR1. 

In conclusion, our study has demonstrated that MUC2 overexpression in ovarian cancer decreases both the progression-free survival rate and the overall survival rate of cancer patients. The detrimental role of MUC2 may be mediated by the imbalanced M1 and M2 polarization of TAMs and could be facilitated by the vigorous PGE_2_ synthesis raised in COX-2^+^ TAMs and cancer cells. Therefore, future studies to identify methods of inhibiting MUC2 expression in ovarian cancer (e.g., using small interfering RNAs) would be worthwhile. In terms of the implications for current clinical oncology, our study indicates that MUC2^++/+++^ ovarian cancer cases should be closely followed up with specific attention to their local immunosuppressive status and that treatment with appropriate immunomodulators, such as COX-2 inhibitors, IL2 and IFNγ, which favor the M1 differentiation of TAMs, should be evaluated.

## Materials and Methods

### Ethics Statement

The protocols for handling paraffin-embedded and liquid nitrogen-cryopreserved ovarian cancer specimens and analyzing patient data were approved by the ethical committees of Renji Hospital, Shanghai Jiaotong University; Obstetrics and Gynecology Hospital, Fudan University; and First Maternity and Infant Health Hospital, Tongji University in Shanghai, China. Written informed consents were signed by each enrolled patient if she was still alive or by her first-degree relative if she has died. All tissue samples were registered by a case number in the database with no patient names or personal information indicated.

### Study population

A total of 102 consecutive pairs of paraffin-embedded and liquid nitrogen-cryopreserved epithelial invasive ovarian cancer specimens diagnosed between January and December 2002 by pathology were enrolled from the three following medical centers in Shanghai, China: Renji Hospital, Shanghai Jiaotong University; Obstetrics and Gynecology Hospital, Fudan University; and First Maternity and Infant Health Hospital, Tongji University. The medical histories of the corresponding patients were carefully reviewed, and all of the relevant data were recorded, including age, height, body weight, gravidity and parity, pleural effusion and ascites status, sites of local and distant metastases, clinical stage (based on the FIGO 2000 diagnostic system [22]), histological type and pathological grade [22] of the cancer and the 5-year follow-up outcomes (survival status: survival or death; disease status: remission or relapse). The three medical centers adopted a standardized ovarian cancer treatment protocol for all patients, that is, (i) an initial cancer status and surgical risk evaluation, (ii) cytoreductive surgery, and (iii) a regular course of postsurgical chemotherapy (taxol + platinum). 

### Immunohistochemical and immunofluorescence analysis

Tissue sections (4 µm thick) were cut onto glass slides, de-waxed using xylene and rehydrated through a gradated series of alcohol [15,26]. Antigen retrieval was conducted using a microwave at medium-high temperature for 15 min and medium-low temperature for 15 min, followed by incubation at room temperature for 2 hours [26]. Mouse anti-human MUC2 (clone 996/1, Abcam, Cambridge, MA, USA, dilution ratio: 1:100) monoclonal antibody or COX-2 (clone 4H12, Abcam, dilution ratio: 1:50) monoclonal antibody and horseradish peroxidase (HRP)-conjugated goat anti-mouse IgG polyclonal antibody (Zhongshan, Beijing, China, dilution ratio: 1:200) were used to label MUC2 and COX-2 in the ovarian cancer specimens and the staining was developed using 3,3’-diaminobenzidine tetrahydrochloride (DAB, Zhongshan). Single or double immunostaining of the TAMs in the ovarian cancer tissues was performed using the following antibodies: rabbit anti-human CD68 polyclonal antibody (product PA5-32330, Thermo, Rockford, IL, USA, dilution ratio: 1:100) or mouse anti-HLA-DR (clone TAL 1B5, Abcam, dilution ratio: 1:50), iNOS (clone 2D2-B2, R&D systems, Minneapolis, MN, USA, dilution ratio: 1:50), CD163 (clone RM3/1, Abcam, dilution ratio: 1:50) or VEGF (clone 5C3.F8, Abcam, dilution ratio: 1:200) monoclonal antibody, together with an HRP-conjugated goat anti-rabbit IgG polyclonal antibody (Zhongshan, dilution ratio: 1:200) and/or an alkaline phosphatase (AP)-conjugated goat anti-mouse IgG multiclonal antibody (Zhongshan, dilution ratio: 1:200). Staining was visualized using DAB or AP-red (Zhongshan), as appropriate. For triple immunostaining, we used CF-488A (Biotium, San Francisco, CA, USA)-labeled rabbit anti-human CD68 polyclonal antibody, CF-633 (Biotium)-labeled mouse anti-human COX-2 monoclonal antibody and a CF-350 (Biotium)-labeled conjugate of one of the following antibodies: mouse anti-human HLA-DR, iNOS, CD163, VEGF or MUC2 monoclonal antibody. For immunohistochemistry, the cell nuclei were counterstained with hematoxylin. For immunofluorescence, the cell nuclei were counterstained with SYTO 40 (Life Technologies, Grand Island, NY, USA). Appropriate mouse IgG isotype control staining was performed, in which the primary antibodies were replaced by irrelevant mouse monoclonal antibodies of the same isotype. Normal nonimmune rabbit serum was used as an IgG control for the rabbit anti-human CD68 polyclonal antibody-labeled sections. The pathological analysis was performed by two independent investigators (gynecological pathologists) who were blinded to the clinical outcomes. To determine the intratumoral TAM densities, ten representative high-power fields (400× magnification) per tissue section were selected using a Leica DM2500 microscope. The number of nucleated cells with positive staining for the phenotype marker(s) in each of the examined cancer tissue areas was counted manually and expressed as cells/mm^2^. The average of the results obtained by the two pathologists was used as the macrophage infiltration density. The expression levels of MUC2 and COX-2 were evaluated semiquantitatively by determining the mean percentage of complete membrane/cytoplasm-stained (the weakly or nuclear-stained cells were excluded) tumor cells in ten microscopic fields at 400× magnification and classifying the values into four groups as 0 (negative), <50 (weakly positive), 50–75 (moderately positive) and >75% (strongly positive). The negative and weakly positive sections were defined as low expression, and the moderately and strongly positive sections were defined as high expression. To validate the effectiveness of the immunohistochemistry-based MUC2 expression level classification, we performed a flow cytometry-based analysis on the cancer cells obtained from the paraffin-embedded tissues (see Materials and Methods S3 in File S1). The results (see Figure S4 in File S2) indicated that the “++” and “+++” immunostained specimens did have MUC2 expression levels significantly higher than those of “-” and “+” cases.

### Statistical analysis

A two-tailed Student's t test was used to compare the numerical data, such as age, height, body weight, gravidity and parity, between the groups of patients with high and low levels of MUC2 expression. ANOVA was used to compare the percentages of total M1 and M2 cells or the percentages of COX-2^+^ M1 and M2 TAMs between the two MUC2 expression groups and to compare the densities of CD68^+^COX-2^+^ TAMs in the cancer islet and stromal regions. The two-sided χ^2^ test was used to analyze the categorical data, such as ascites, lymph node metastasis, distant metastasis, clinical stage, histotype and pathological grade. Pearson’s product-moment correlation coefficient was applied to estimate the relationship between the COX-2^+^ cancer cell density and the COX-2^+^ TAM density in the enrolled specimens. A Kaplan-Meier survival analysis was used to evaluate the impacts of several important pathological parameters, such as age, BMI, ascites, peritoneal and lymphatic metastasis, residual site, clinical stage, histotype, grade, MUC2 expression level, M1/M2 ratio, COX-2^+^ cancer cell density and COX-2^+^ TAM density, on the progression-free survival and overall survival rates, whereas the log-rank test was used to establish significant differences. The multivariate Cox regression model was used to analyze the hazard ratios (HRs) of the aforementioned prognostic factors and to examine their independence. The analyses were performed with SPSS 13.0 software (IBM, Armonk, New York, USA), and p <0.05 was considered statistically significant.

## Supporting Information

File S1 **Supplementary Materials and Methods.** Materials and methods S1 describes the methods used for the collection of an additional population of patients with benign ovarian tumors. Materials and Methods S2 describes the methods used for performing the PGE_2_ ELISA in clinical samples. Materials and Methods S3 describes the flow cytometry method used to validate the effectiveness (objectivity) of the immunohistochemistry analysis for determining the MUC2 expression levels in specimens.
(PDF)

File S2 **Supplementary Figures.** Figure S1 shows the comparison result of the percentages of cases with different MUC2-immunostaining levels in patients with benign and malignant ovarian tumors. Figure S2 shows the comparison results of the PGE2 concentrations in cancer tissues from the high MUC2 expression group and the low MUC2 expression group as well as in various histotypes of ovarian cancers. Figure S3 shows the Kaplan–Meier 5-year PFS and OS curves for ovarian cancer patients stratified by eight pathological parameters. Figure S4 shows the results of the flow cytometry-based validation of the immunohistochemically stained “++“/”+++” and “-“/”+” cases. Figure S5 shows representative microscopic fields of CD68^+^ TAMs in different histotypes of ovarian cancer. Figure S6, Shows the results of a comparison of the CD68+ TAM densities among various ovarian cancer histotypes. Figure S7 shows the results of a comparison of the percentages of different M1 and M2 cell subsets (i.e., M1/M2 distribution patterns) among all the TAMs for different histotypes of ovarian cancer.
(PDF)

Table S1 **The comparison results of selected demographic and pathological characteristics of the patients with benign and malignant ovarian tumors.**
(PDF)
