# Supplementary material for: High MUC2 Expression in Ovarian Cancer Is Inversely Associated with the M1/M2 Ratio of Tumor-Associated Macrophages and Patient Survival Time
Source: PLoS One. 2013 Dec 6;8(12):e79769. doi: 10.1371/journal.pone.0079769 (PMC3855613; doi:10.1371/journal.pone.0079769)
Supplement: File S1 — Supplementary Materials and Methods. Materials and methods S1 describes the methods used for the collection of an additional population of patients with benign ovarian tumors. Materials and Methods S2 describes the methods used for performing the PGE2 ELISA in clinical samples. Materials and Methods S3 describes the flow cytometry method used to validate the effectiveness (objectivity) of the immunohistochemistry analysis for determining the MUC2 expression levels in specimens. (PDF) [file pone.0079769.s001.pdf]

Supplementary materials and methods

Materials and Methods S1

Additional study population

To investigate the MUC2 expression statuses in benign ovarian tumors, we examined paraffin-embedded specimens from 33 patients with benign epithelial ovarian tumors, such as serous and mucinous cystoadenomas, from the aforementioned three medical centers. The pathological diagnoses of these patients were all reached during the same time period as those of the ovarian cancer cases. Informed consent was also provided by all of the enrolled patients. This additional protocol was also approved by the ethics committees of Renji Hospital, Obstetrics and Gynecology Hospital and First Maternity and Infant Health Hospital, Shanghai, China.

Materials and Methods S2

Enzyme-linked immunosorbent assay (ELISA)

To estimate the PGE2 concentrations in cancer tissues, a 1-g sample was resected from the liquid nitrogen-cryopreserved (-196 ℃) cancer specimen for each patient. The obtained sample was thawed at room temperature, placed in 1 mL of phosphate-buffered saline (PBS, 137 mM NaCl, 2.7 mM KCl, 4.3 mM Na_2HPO_4, 1.4 mM KH_2PO_4, pH 7.4) and then thoroughly homogenized on ice within 10 min. The homogenate was then centrifuged at 12,000 rpm for 5 min, and the supernatant was collected. For each assay, 100 μL of the supernatant of the homogenized cancer tissue was added to a well of the 96-well ELISA plate from the Human PGE_2 ELISA kit (product no. CSB-E07965 h, Cusabio, Shanghai, China) and sequentially treated with detection antibodies and chromogenic substrate according to the manufacturer’s instructions. The reaction was terminated by adding 50 μL of 1 M H_2SO_4 to the well, and the optical density was read at 450 nm. The PGE_2 concentration was determined based on a standard curve that was produced using the serially diluted reference samples provided in the PGE_2 ELISA kit and presented as μg/L.

Materials and Methods S3

Flow cytometry

Flow cytometry was used to validate the effectiveness (objectivity) of immunohistochemistry for the determination of the MUC2 expression level in specimens. To prepare a cell suspension, a 30-μm paraffin-embedded section was dewaxed in xylene and rehydrated through a series of alcohols (100%, 95%, 70% and 50%) in distilled water. The section was treated for 60 minutes at 37℃ in a 1 mg/ml solution of collagenase IV (Sigma, St. Louis, MO, USA). The obtained cell suspension was filtrated through a 35-μm pore nylon gauze. To avoid non-specific binding to Fc receptors, especially those expressed on the membranes of intratumoral blood cells, the suspensions were treated with human immunoglobulins (Sangon, Shanghai, China). Then, cells were stained using a fluorescein isothiocyanate (FITC)-conjugated mouse anti-human MUC2 antibody (clone 996/1, Abcam), a phycoerythrin (PE)-conjugated anti-human pan-Keratin antibody (clone 80, Abcam) and propidium iodide (PI) and analyzed using a Beckman Coulter FC500 MPL flow cytometer in a three-color mode.

FITC-conjugated and PE-conjugated mouse anti-rabbit IgG antibodies (clone 2A9, Abcam) were used as negative controls. PI-negative cells were gated and excluded from the flow cytometry analysis. Compared with cells stained with PE-conjugated mouse anti-rabbit IgG antibody, the cells detected with higher levels of PE fluorescence were defined as Keratin positive (ovarian cancer). The Keratin-positive cells were analyzed for the mean fluorescence intensity of FITC to determine the MUC2 expression level, which was controlled by cells stained with FITC-conjugated mouse anti-rabbit IgG antibody. Typically, 10,000 to 20,000 events were collected in the flow cytometry analysis.
