# Supplementary material for: High MUC2 Expression in Ovarian Cancer Is Inversely Associated with the M1/M2 Ratio of Tumor-Associated Macrophages and Patient Survival Time
Source: PLoS One. 2013 Dec 6;8(12):e79769. doi: 10.1371/journal.pone.0079769 (PMC3855613; doi:10.1371/journal.pone.0079769)
Supplement: File S2 — Supplementary Figures. Figure S1 shows the comparison result of the percentages of cases with different MUC2-immunostaining levels in patients with benign and malignant ovarian tumors. Figure S2 shows the comparison results of the PGE2 concentrations in cancer tissues from the high MUC2 expression group and the low MUC2 expression group as well as in various histotypes of ovarian cancers. Figure S3 shows the Kaplan–Meier 5-year PFS and OS curves for ovarian cancer patients stratified by eight pathological parameters. Figure S4 shows the results of the flow cytometry-based validation of the immunohistochemically stained “++“/”+++” and “-“/”+” cases. Figure S5 shows representative microscopic fields of CD68+ TAMs in different histotypes of ovarian cancer. Figure S6, Shows the results of a comparison of the CD68+ TAM densities among various ovarian cancer histotypes. Figure S7 shows the results of a comparison of the percentages of different M1 and M2 cell subsets (i.e., M1/M2 distribution patterns) among all the TAMs for different histotypes of ovarian cancer. (PDF) [file pone.0079769.s002.pdf]

Supplementary figures

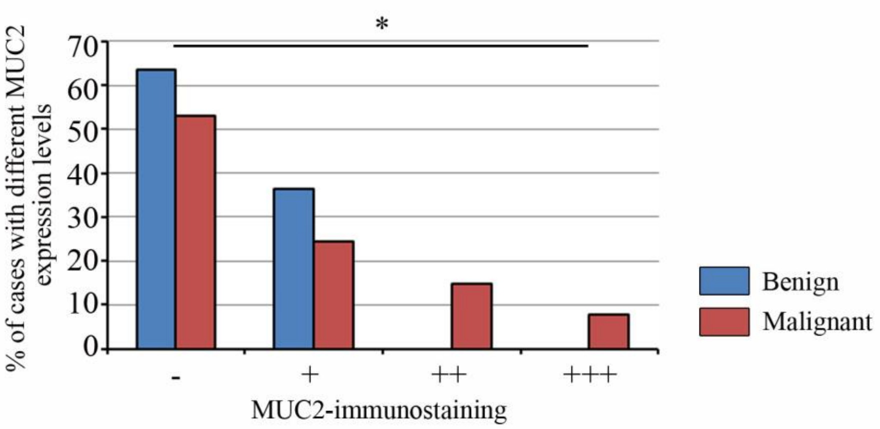

Figure S1. Comparison of the percentages of cases with different MUC2-immunostaining levels in patients with benign and malignant ovarian tumors. *, p<0.05, χ^2 test.

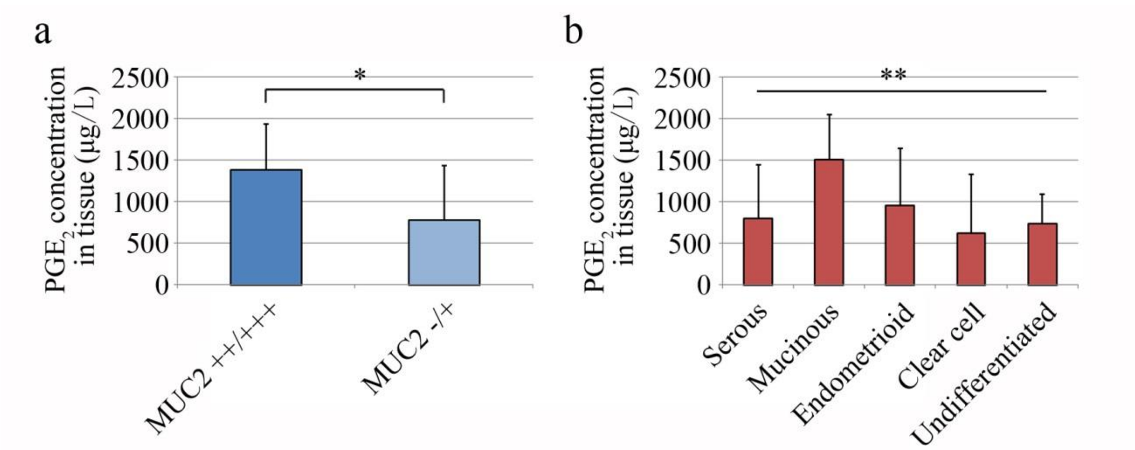

Figure S2. Comparison of the PGE_2 concentrations in cancer tissues from the high MUC2 expression group and the low MUC2 expression group (a) as well as in various histotypes of ovarian cancers (b). *, p<0.05, Student's t test. **, p<0.05, ANOVA.

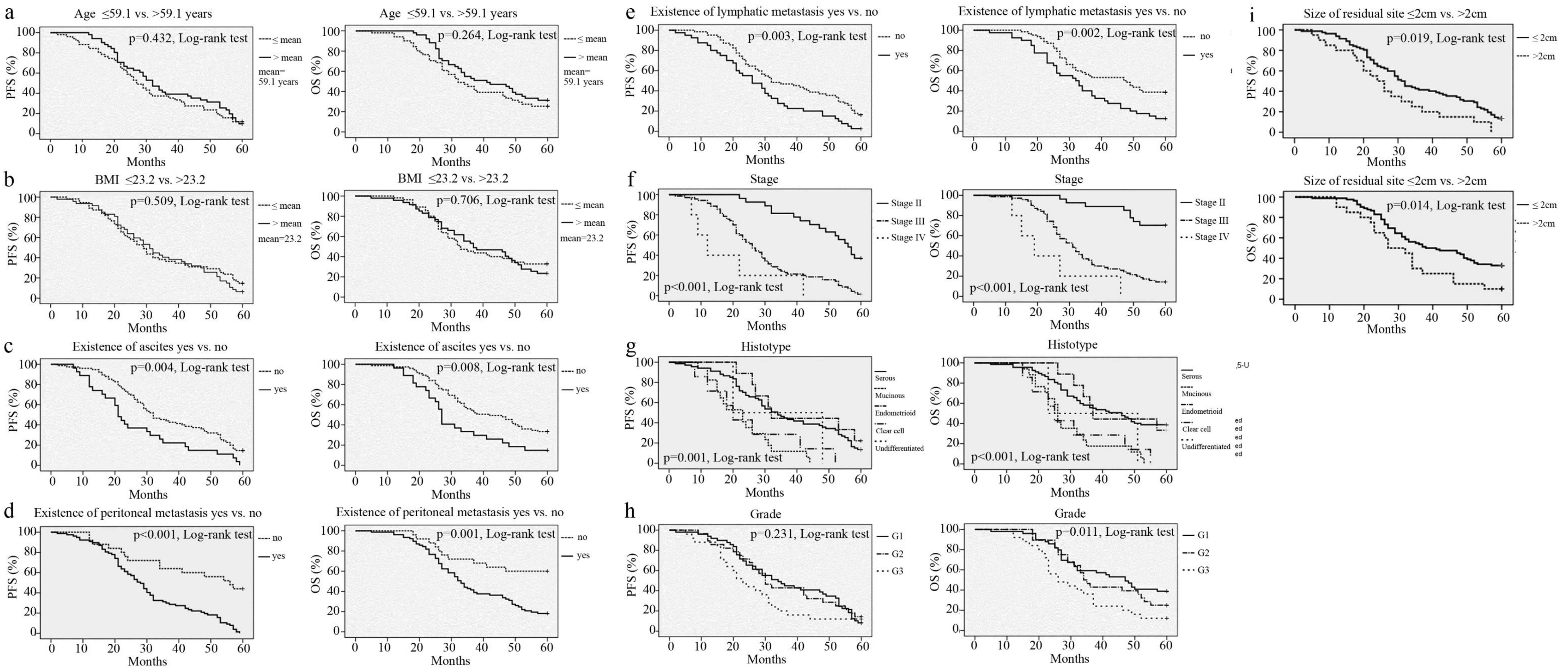

Figure S3. Kaplan–Meier 5-year PFS and OS curves for ovarian cancer patients stratified by eight pathological parameters: age (a), BMI (b), ascites (c), peritoneal metastasis (d), lymphatic metastasis (e), stage (f), histotype (g) grade (h) and residual site (i). PFS, progression-free survival. OS, overall survival.

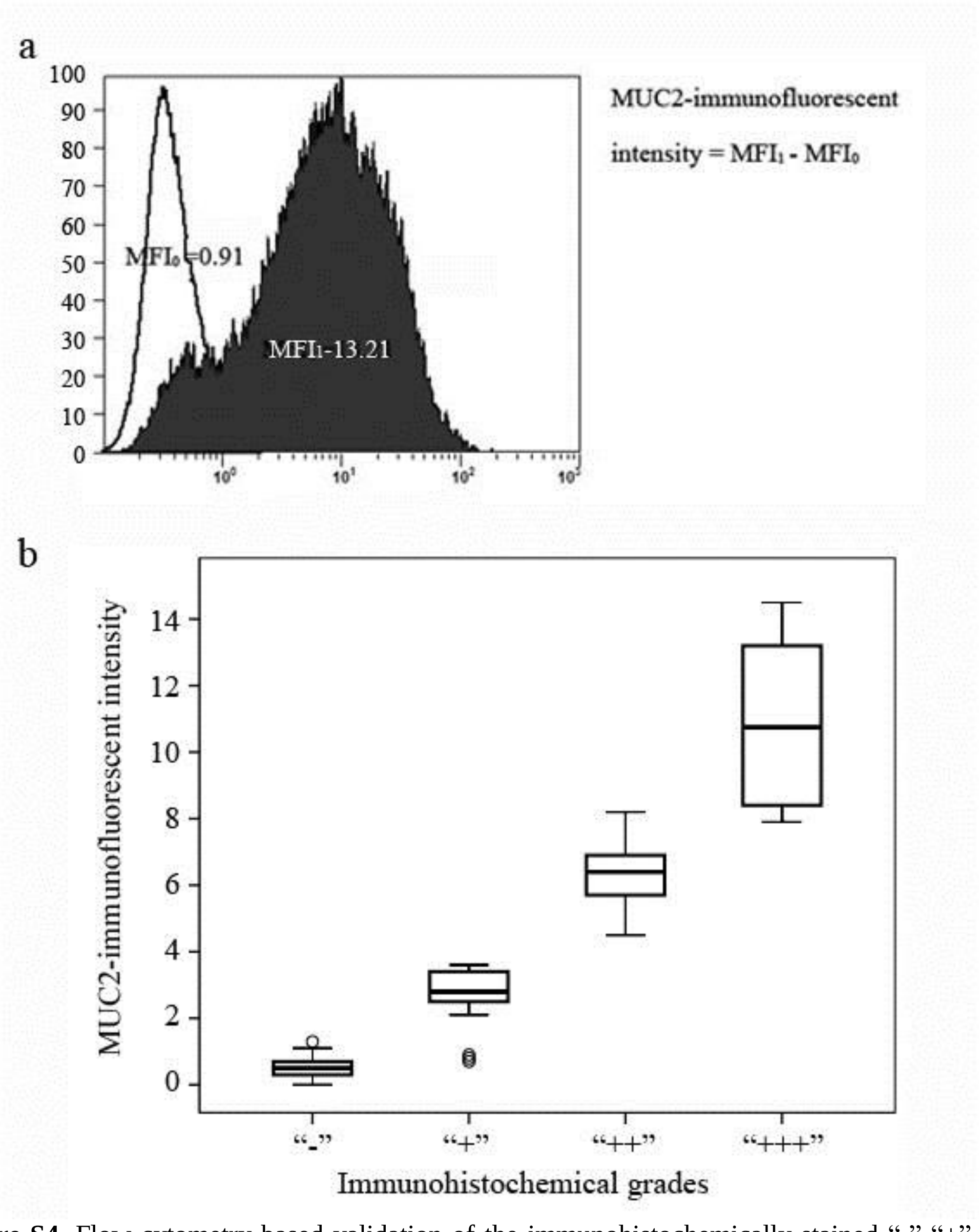

Figure S4. Flow cytometry-based validation of the immunohistochemically stained "-" "+" "++" and "+++" cases. (a) The MUC2-immunofluorescent intensity was calculated by MFI_1 minus MFI_0. MFI_0: The mean fluorescent intensity of non-specific antibody (FITC-conjugated rabbit anti-mouse IgG antibody)-labeled cancer cells. MFI_1: The mean fluorescent intensity of MUC2 antibody-labeled cancer cells. (b) The "-" "+" "++" and "+++" cases showed significant differences between one another in terms of their mean MUC2-immunofluorescent intensities. $p<0.001$, ANOVA.

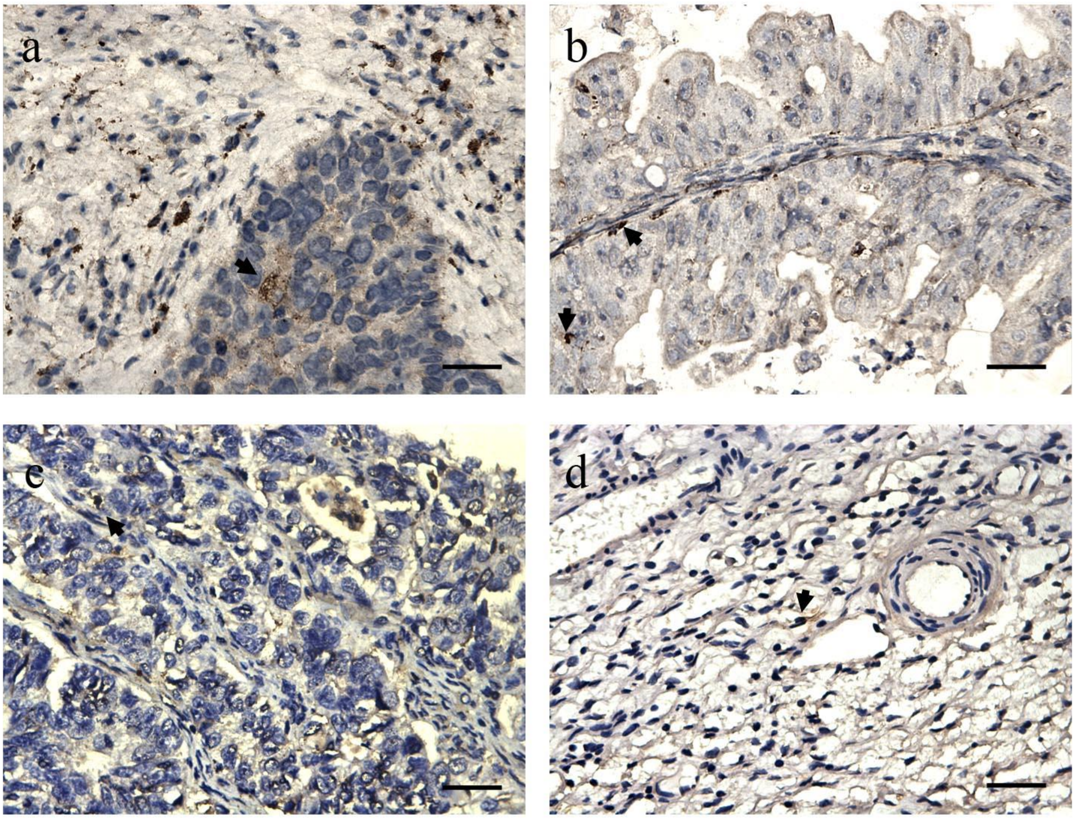

Figure S5. $CD68^+$ TAMs in ovarian cancer tissues. (a) Serous ovarian cancer. (b) Mucinous ovarian cancer. (c) Endometrioid ovarian cancer. (d) Clear cell ovarian cancer. Bar: 10μm. Arrow: $CD68^+$ TAM.

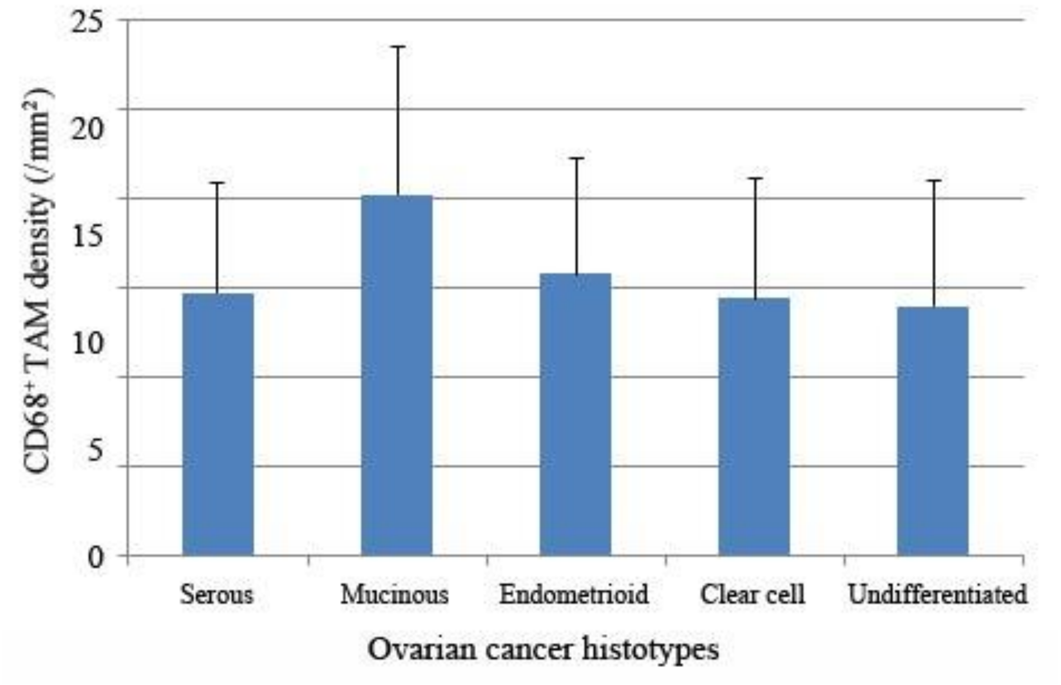

Figure S6. $CD68^+$ TAM densities in different histotypes of ovarian cancer. (p=0.055, ANOVA)

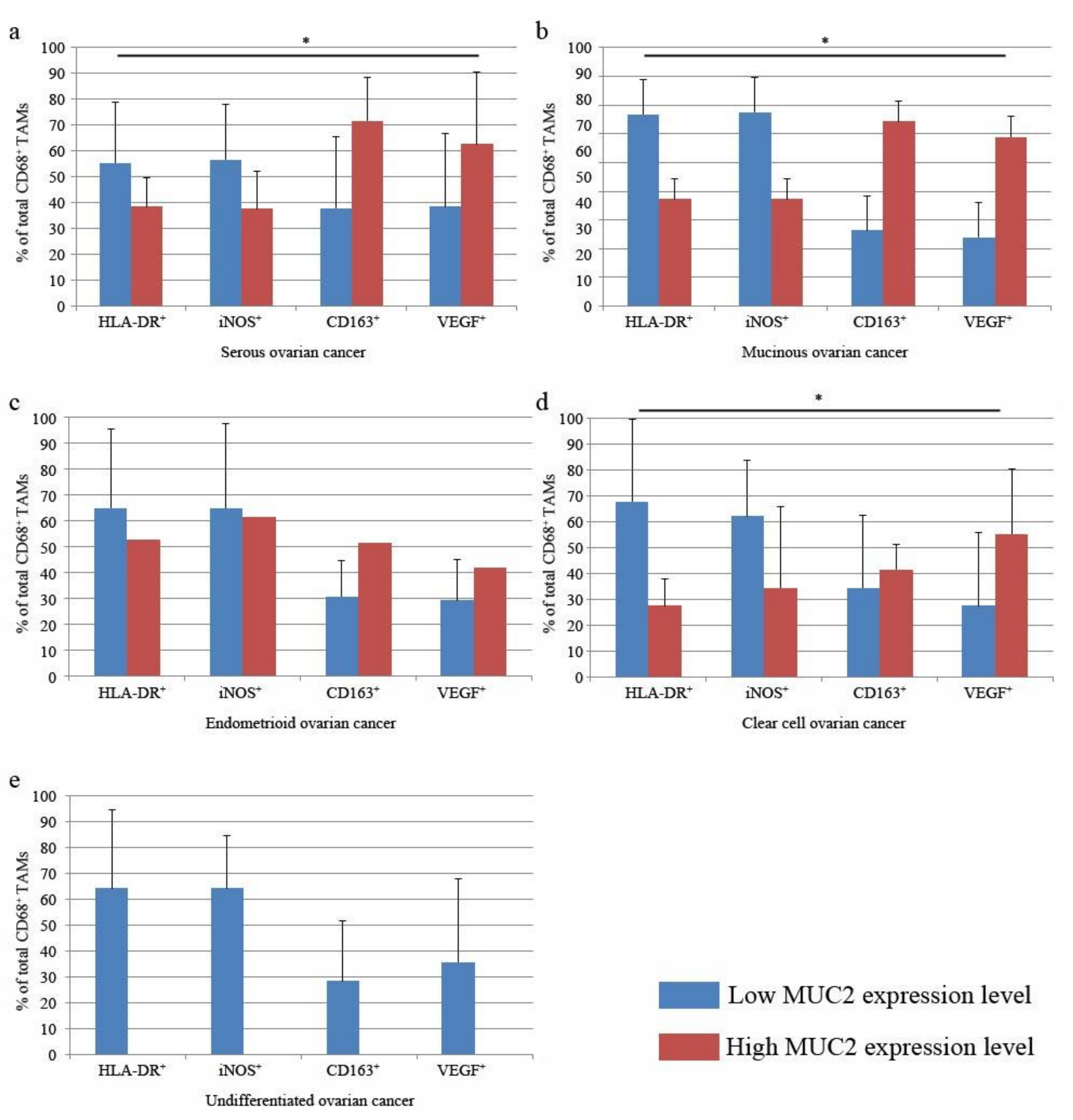

Figure S7. Comparison of the percentages of different M1 and M2 cell subsets (i.e., M1/M2 distribution patterns) among all the TAMs according to ovarian cancer histotypes. (a) Serous ovarian cancer. (b) Mucinous ovarian cancer. (c) Endometrioid ovarian cancer. (d) Clear cell ovarian cancer. (e) Undifferentiated ovarian cancer. *: $p<0.05$, ANOVA. Because only one case and no case were found in the high MUC2 expression level groups of the endometrioid and undifferentiated ovarian cancer cases, respectively, we have not performed any statistical analyses on the M1 and M2 distribution patterns for these two cancer histotypes.
