# Supplementary material for: High MUC2 Expression in Ovarian Cancer Is Inversely Associated with the M1/M2 Ratio of Tumor-Associated Macrophages and Patient Survival Time
Source: PLoS One. 2013 Dec 6;8(12):e79769. doi: 10.1371/journal.pone.0079769 (PMC3855613; doi:10.1371/journal.pone.0079769)
Supplement: Table S1 — The comparison results of selected demographic and pathological characteristics of the patients with benign and malignant ovarian tumors. (PDF) [file pone.0079769.s003.pdf]

**Supplementary Table**

**Table S1. A comparison of selected demographic and pathological characteristics of the patients with benign and malignant ovarian tumors ***

| Characteristics | Benign ovarian tumor (n=33) | Malignant ovarian tumor (n=102) | p value † |
|---|---|---|---|
| Demographics: | | | |
| Age (years) | 45.7 ±11.3 | 59.3 ±6.2 | <0.001 § |
| Gravidity | 3.1 ±1.8 | 4.1 ±1.1 | <0.001 § |
| Parity | 0.8 ±0.7 | 1.1 ±0.4 | 0.005 § |
| BMI | 23.0 ±1.5 | 23.2 ±2.4 | 0.803 |
| Oral contraceptive use >1/2 year | 3 (9.1) | 9 (8.8) | 0.963 |
| Smoking >1/2 year | 8 (24.2) | 17 (16.7) | 0.330 |
| Alcohol abuse >1/2 year | 2 (6.1) | 2 (2.0) | 0.227 |
| | | | |
| Pathology: | | | |
| Histotype | | | 0.032 § |
| Serous | 31 (93.9) | 67 (65.7) | |
| Mucinous | 2 (6.1) | 17 (16.7) | |
| Endometrioid | 0 | 9 (8.8) | |
| Clear cell | 0 | 7 (6.9) | |
| Undifferentiated | 0 | 2 (2.0) | |
| MUC2 immunostaining | | | 0.026 § |
| - | 21 (63.6) | 54 (52.9) | |
| + | 12 (36.4) | 25 (24.5) | |
| ++ | 0 (0) | 15 (14.7) | |
| +++ | 0 (0) | 8 (7.8) | |

* Data are presented as the mean values ±standard deviation or the number (percentage)

† The two-tailed Student's t-test or $\chi^2$ test was used, as appropriate.

§ Statistical significance.
